# Predictors of in-hospital mortality and complications in very elderly patients undergoing emergency surgery

**DOI:** 10.1186/1749-7922-9-43

**Published:** 2014-07-07

**Authors:** Shaheed Merani, Judd Payne, Raj S Padwal, Darren Hudson, Sandy L Widder, Rachel G Khadaroo

**Affiliations:** 1 Department of Surgery, Faculty of Medicine and Dentistry, University of Alberta, Edmonton, Alberta, Canada; 2 Department of Medicine, University of Alberta, Edmonton, Alberta, Canada; 3 Division of Critical Care Medicine, Faculty of Medicine and Dentistry, University of Alberta, Edmonton, Alberta, Canada; 4 Department of Surgery, University of Alberta, 2D Walter Mackenzie Center, 8440-112 St. NW, Edmonton, Alberta T6G 2B7, Canada

**Keywords:** Elderly, Acute care, Emergency, Surgery, Morbidity, Mortality

## Abstract

**Introduction:**

With the increasing aging population demographics and life expectancies the number of very elderly patients (age ≥ 80) undergoing emergency surgery is expected to rise. This investigation examines the outcomes in very elderly patients undergoing emergency general surgery, including predictors of in-hospital mortality and morbidity.

**Methods:**

A retrospective study of patients aged 80 and above undergoing emergency surgery between 2008 and 2010 at a tertiary care facility in Canada was conducted. Demographics, comorbidities, surgical indications, and perioperative risk assessment data were collected. Outcomes included length of hospitalization, discharge destination, and in-hospital mortality and morbidity. Multivariable logistic regression was used to identify predictors of in-hospital mortality and complications.

**Results:**

Of the 170 patient admissions, the mean age was 84 years and the in-hospital mortality rate was 14.7%. Comorbidities were present in 91% of this older patient population. Over 60% of the patients required further services or alternate level of care on discharge. American Society of Anesthesiologist Physical Status (ASA) Classification (OR 5.30, 95% CI 1.774-15.817, p = 0.003) and the development of an in-hospital complications (OR 2.51, 95% CI 1.210-5.187, p = 0.013) were independent predictors of postoperative mortality. Chronological age or number of comorbidities was not predictive of surgical outcome.

**Conclusions:**

Mortality, complication rates and post-discharge care requirements were high in very elderly patients undergoing emergency general surgery. Advanced age and medical comorbidities alone should not be the limiting factors for surgical referral or treatment. This study illustrates the importance of preventing an in-hospital complication in this very vulnerable population. ASA class is a robust tool which is predictive of mortality in the very elderly population and can be used to guide patient and family counseling in the emergency setting.

## Introduction

It is estimated that the majority of people born in developed nations during the 21^st^ century will live to their 100^th^ birthdays [1]. Both the increased number of elderly and the inherent complexity of their health have resulted in increased demands on the health care system [1-5]. Comparative studies across nations have suggested that increased survival to the highest ages is associated with worse health [1]. Overall, the current population will be living longer with more health problems than in the past.

The very elderly (age ≥ 80 years) often suffer from frailty. Frailty is associated with advanced age, but is also influenced by comprehensive determinants including medical comorbidity, nutritional status, mental health, social support, and cognition [6]. Neither a single definition nor measure of frailty exists; however, there is consensus that very elderly individuals have an increased risk of adverse outcomes from physiological stress and disease.

A growing body of evidence on the outcome of elective operative management of very elderly patients has become available over the last decade [6-12]. However, there are limited data on the outcome of very elderly patients undergoing emergency general surgical procedures [6,13-15]. While elective surgical care affords the benefit of comprehensive geriatric assessment and the pre-operative optimization of comorbid states, emergency surgery differs in that there is limited time for information collection (including goals of care). The baseline health, mental, and social status of elderly patients who present with acute surgical emergencies is often unknown and comorbidities under recognized. The absence of this information exacerbates the vulnerability of these patients to known insults which occur during hospitalization [16]. Post-operative care itself has traditionally been a source of such insults including fasting for gastrointestinal healing, polypharmacy, immobility, nasogastric tubes, and bladder catheterization. These, in turn, place surgical patients at higher risk of complications including delerium [8].

The purpose of this study is to characterize the very elderly population, who received emergency general surgery, and examine their surgical outcomes including identification of factors associated with in-hospital mortality and morbidity. We hypothesized that the number of medical comorbidities and American Society of Anesthesiologist Physical Status Classification (ASA class) would be the strongest predictors of poor outcomes.

## Materials & methods

A retrospective cohort study was conducted on very elderly patients undergoing emergency general surgery at the University of Alberta Hospital, a tertiary care academic teaching hospital in Edmonton, Alberta, Canada between 2008 and 2010.

Inclusion criteria included patients who had an age of 80 years or older and at least one emergency general surgical procedure during admission. We defined emergency surgery as an operative procedure that was meant to prevent morbidity or mortality, not booked from an outpatient clinic (elective basis), and required an unplanned operation on their admission to hospital.

Patient demographics including age, sex, weight, height, pre-hospitalization medication use and comorbidities were collected. Additionally, operative data including anesthesiologist assigned ASA class, Comorbidity-Polypharmacy Score (CPS) (which combines the number of pre-illness medications with the number of comorbidities to estimate the severity of comorbid condition [17]), operative procedure performed, and surgical diagnoses were collected. Clinical outcomes measured included in-hospital complications, length of hospital stay, in-hospital mortality, and discharge location. The University of Alberta Human Research Ethics Board approved this research.

Data was collected using a Microsoft Access database, and statistical analysis was performed with SPSS 17.0. Frequencies and percentages were tabulated for categorical and ordinal variables; means and standard deviations calculated for continuous variables. The statistical association between categorical variables was studied with chi-square analysis. Binary logistic regression analysis was used to identify predictors of in-hospital mortality and complications. A multi-variate model was built using age, gender, BMI, number of pre-hospitalization medications and comorbidities, ASA class, and number of in-hospital complications as factors entered in a single step. A p-value of < 0.05 was considered evidence of an association not attributable to chance, and therefore of statistical significance.

## Results

### Patient demographics, diagnoses, and operative procedures

During three years studied (2008-2010) a total of 170 patients aged 80 or above were admitted for emergency general surgery. The mean age was 84.1 years, over half were male (51.2%), and the average BMI was 24.8 kg/m^2^ (Table 1).

**Table 1:** Patient admission characteristics and comorbidities

	**n (%)**
**Age** (years) Mean = 84.1 (SD = 3.6)	
80-84	105 (61.8%)
85-90	50 (29.4%)
≥ 90	15 (8.8%)
**Sex**	
Female	83 (48.8%)
**BMI** (kg/m^2^) Mean = 24.8 (SD = 4.6)	
< 18.5 (Underweight)	13 (7.6%)
18.5-25 (Normal weight)	74 (43.5%)
25-30 (Overweight)	53 (31.2%)
> 30 (Obese)	19 (11.2%)
**ASA class**	
1E	1 (0.7%)
2E	11 (8.2%)
3E	78 (58.2%)
4E	44 (32.8%)

Comorbid illness was present in 91.2% of elderly patients in this cohort. The most common were hypertension, respiratory disease (including COPD), diabetes, hypothyroidism, and heart failure (Table 2). Correspondingly, 89% of patients were using at least one home medication prior to hospitalization. The most common medications used were angiotensin converting enzyme inhibitors, anti-platelet agents, beta-blockers, statins, and diuretics (Table 2). Median ASA class was 3E (58.2% of patients) (Table 1). Median CPS score was 6 (range of 0 to 14).

**Table 2:** Patient comorbidities: total comorbidity number, medication use, ASA class, and CPS

	**n (%)**
**Comorbidity**	
Hypertension	112 (65.9%)
Respiratory disease (including COPD)	44 (25.9%)
Diabetes	34 (20%)
Hypothyroid	33(19.4%)
Heart failure	29 (17.1%)
Osteoarthritis	26 (15.3%)
Osteoporosis	23 (13.5%)
Smoking history	19 (11.2%)
Stroke with residual deficit	7 (4.1%)
Myocardial infarction (within last 6 months)	7 (4.1%)
**Total number of comorbidities**	
None	15 (8.8%)
1-2	95 (55.9%)
3-5	58 (34.1%)
> 5	2 (1.2%)
**Number of home medications**	
None	19 (11.2%)
1-2	37 (21.8%)
3-5	81 (47.6%)
> 5	33 (19.4%)
**Home medication use**	
ACE inhibitor	73 (42.9%)
Anti-platelet agent	73 (42.9%)
Beta-blocker	66 (38.8%)
Statin	62 (36.5%)
Diuretics	54 (31.8%)
Calcium channel blocker	45 (26.5%)
Anti-coagulant	42 (24.7%)
**CPS**	
0-3	44 (25.9%)
4-7	80 (47.1%)
8-10	36 (21.2%)
> 10	10 (5.9%)

The majority of emergency general surgical procedures were for colon resection (22.9%), small bowel resection (19.4%) or laparotomy (15.9%) followed by cholecystectomy (10.6%) (Table 3).

**Table 3:** Diagnoses and procedures performed

	**n (%)**
**Operative procedure**	
Colon (Laparotomy for resection or diversion)	39 (22.9%)
Small Bowel (Laparotomy for adhesions or resection)	33 (19.4%)
Laparotomy (other)	27 (15.9%)
Cholecystectomy	18 (10.6%)
Hernia – Incarcerated/Strangulation	15 (8.8%)
Duodenal Bleed/Perforation	9 (5.3%)
**Primary diagnosis**	
Small Bowel Obstruction	25 (14.7%)
Hernia	20 (11.8%)
Cholelithiasis (Complicated)	17 (10%)
Colon Cancer	14 (8.2%)
Duodenal Ulcer	13 (7.6%)
Appendicitis	9 (5.3%)
Bowel Ischemia	9 (5.3%)
Colon Obstruction	9 (5.3%)
Colon Perforation	8 (4.7%)
Gastrointestinal Bleed	6 (3.5%)

Common diagnoses and procedures performed on admitted patients.

### In-hospital complication, mortality, and length of stay

In-hospital complications were experienced by over a fifth of patients (Table 4). The most common complications were pulmonary in nature (16.5% of patients) including respiratory failure (requiring intensive care unit support), pneumonia, and pulmonary embolism. Other common complications included both surgical (post-operative bleeding, wound infection and dehiscence), and medical (acute or acute-on-chronic renal failure).

**Table 4:** Complications, mortality, length of stay, and disposition following surgery

	**n (%)**
**Complication**	
Respiratory failure (requiring intubation)	12 (7.1%)
Bleeding	11 (6.5%)
Renal Failure	10 (5.9%)
Sepsis	9 (5.3%)
Wound Complication	8 (4.7%)
PE	3 (1.8%)
Stroke	2 (1.2%)
**Total number of complications**	
0	135 (79.4%)
1-2	30 (17.6%)
3-5	5 (2.9%)
**Mortality**	25 (14.7%)
**Length of Stay** (Median 14 days)	
< 7 days	36 (21.2%)
8-14 days	52 (30.6%)
15-30 days	45 (26.5%)
31-90 days	30 (17.6%)
> 90 days	6 (3.5%)
**Disposition** (n = 145)	
Home	78 (53.8%)
Without additional services	54 (37.2%)
With homecare services	24 (16.7%)
Rehabilitation/home hospital	54 (37.2%)
Assisted Living/long term care	9 (6.2%)
Other	4 (2.8%)

A total of 25 of very elderly patients receiving emergency surgery died in the hospital (14.7% mortality). There was lower mortality in the octogenarian group (12.9%) compared with 33% in the nonagenarian group, while not statistical significant this may be reflective of the relatively small numbers in the groups (Table 1, p = 0.08).

The median length of stay was 14 days (range 1 to 164 days). Twenty one percent of patients remained in hospital for greater than 30 days (not including any post-discharge admission to a transition or rehabilitation facility). Of the patients who were discharged from hospital, 62% required residential health services beyond their admission (transfer to another hospital, assisted care facility, rehabilitation center, or home-care nursing). Over a third of patients were discharged home without services.

### Predictors of in-hospital morbidity and complications

**Figure 1:**
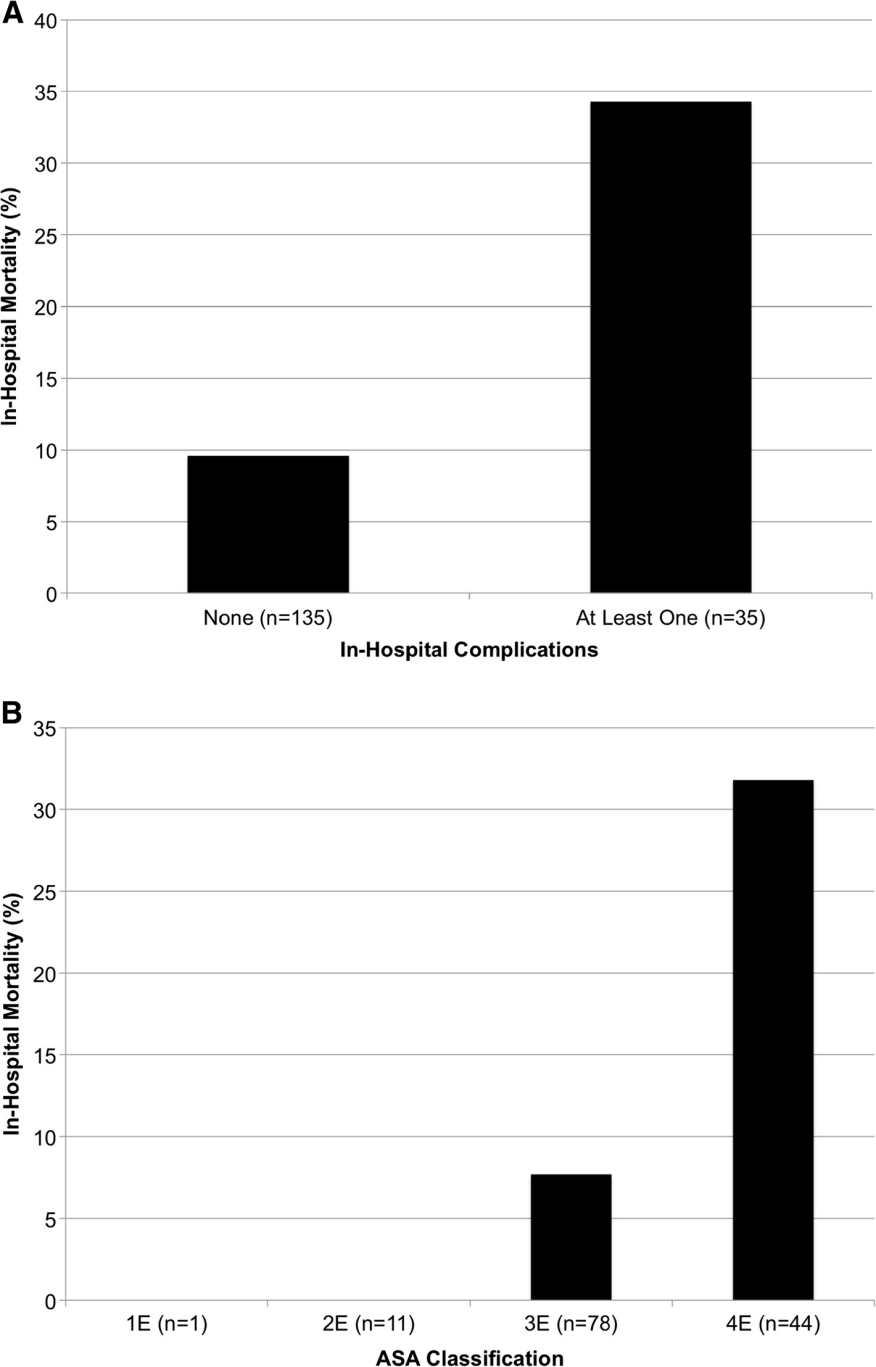
**Multivariable Logistic regression analysis demonstrated statistically significant factors predictive of in-hospital mortality.** Development of in-hospital complication is predictive of in-hospital mortality **(A)**, and increasing ASA class is predictive of in-hospital mortality **(B)**.

Multivariable logistic regression analysis was used to identify variables associated with in-hospital mortality (Table 5). Of these, ASA class (OR 5.30, 95% CI 1.774-15.817, p = 0.003) and in-hospital complications (OR 2.51, 95% CI 1.210-5.187, p = 0.013) were statistically significantly predictive of in-hospital mortality (Figure 1). Majority of the patients were ASA class 3 (n = 78, 58%). The death rate for each ASA class were 1 (0%), 2 (0%), 3 (7.7%) and 4 (31.8%). The number of comorbidites, age, or CPS score was not predictive of mortality. The regression model to identify those patients at higher risk of at least one in-hospital complication (Table 6) did not identify any statistically significant covariates.

**Table 5:** Factors associated with in-hospital mortality - multivariable logistic regression analysis

**Factor**	**B**	**p-value**	**OR**	**95% CI for OR**	
				**Lower**	**Upper**
Age	.061	.436	1.063	.912	1.239
Sex (Female)	.407	.488	1.502	.476	4.743
BMI	.019	.755	1.019	.904	1.150
Medications	-.118	.425	.889	.665	1.188
Comorbidities	.388	.093	1.474	.938	2.318
ASA class	1.667	.003*	5.297	1.774	15.817
Complications	.918	.013*	2.505	1.210	5.187

*p < 0.05.

**Table 6:** Factors associated with in-hospital morbidity - multivariable logistic regression analysis

**Factor**	**B**	**p-value**	**OR**	**95% CI for OR**	
				**Lower**	**Upper**
Age	-.096	.254	.908	.770	1.071
Sex (Female)	.051	.919	1.053	.392	2.828
BMI	.012	.826	1.013	.906	1.132
Medications	.118	.348	1.125	.879	1.440
Comorbidities	-.210	.304	.810	.543	1.210
ASA class	.409	.325	1.506	.667	3.399

## Conclusion

By the year 2040 it is estimated that greater than 25% of the population will be seniors [18]. The rapid growth of the aging population has prompted the necessity for a better understanding of the needs and outcomes of elderly patients undergoing emergency surgery. The present study demonstrates that the majority of patients aged 80 or above admitted for emergency general surgery had pre-existing co-morbidity, were taking one or more medications, and had functional limitations of their illness (as demonstrated by an ASA class of 3E or above). Over sixty percent of the patients in this study required additional healthcare services beyond their admission. There is relatively good long-term survival in this very elderly population where we found fifty percent alive on our three years post-surgery follow-up [19]. From a system perspective, early resource utilization planning can occur if we better understand this population’s predicted demand for acute care beds and longer term need for appropriate supportive care, alternate level of care, and rehabilitation or transition beds.

There is a paucity of studies examining emergency surgery in elderly patients, which makes it difficult to determine outcomes in this patient population. In ambulatory medical practice and elective surgery, adverse outcomes are associated with frailty measures including loneliness, cognitive impairment functional limitations, poor nutritional status, and depression [6,7]. In the Reported Edmonton Frail Scale (REFS) as well as other frailty scales, measures of general health (comorbidities and medications) constitute only a very small portion of the composite frailty [20], however, in the emergency setting, it is a challenge to perform a comprehensive geriatric assessment of frailty.

Other scoring systems to estimate outcomes and mortality in elderly surgical patients include the Acute Physiology and Chronic Health Evaluation II (APACHE II) score [21]. However, the APACHE II score can be difficult to apply to the typical emergency surgery patient since it is predictive only for patients in the critical care setting. As well, an arterial blood gas is not typically part of the pre-operative work-up. The APACHE II is a score that is applied within the first 24 hours to a critically ill patient; therefore, it also does not take into account the physiological insults and complications that an elderly patient may experience at a later time. By contrast the ASA classification, initially described by Saklad et al. 1941, can be quickly determined on admission [22]. It has been shown to be predictive of complications and mortality in a global surgical cohort [23]. Our study reinforces that higher ASA class is associated with mortality following emergency general surgery in the elderly. While anesthesia providers often use this score our study demonstrates the value for surgeons using the ASA classification for preoperative risk stratification and discussions.

There may be reluctance by physicians to refer patients for surgical treatment due to advanced age and medical co-morbidities. However, our findings show there was no clear relationship between chronologic age or number of comorbidities with postoperative outcome (morbidity or mortality) after multivariable adjustment. Therefore, age or comorbidities alone should not be the limiting factors for surgical referral or treatment. For most of these surgically treated illnesses, withholding operative care will result in death. Our results indicate markedly higher mortality with rising ASA class. Specifically patients with ASA 4 (severe systemic disease that is a constant threat to life) had the highest risk of death at 33%. Which means surgeons can use this information preoperatively to give estimates of death and morbidity to patients and families.

Our analysis suggests that chronological age alone in the cohort of patients aged 80 and above is not a robust measure of outcome. This could be due to a lack of statistical power. However, it may also be that chronological age is not a major predictor of mortality once more important predictors, such as baseline physical health (ASA class), is accounted for. Or potentially there may even be a ceiling effect of age wherein age alone does not affect morality in the very elderly population.

Although it is always desirable to prevent complications, it is impossible to perform surgery that is complication free. Surgical complications in this group involve a complex interrelationship between baseline vulnerability and precipitating insults occurring during hospitalization [16]. Emergency abdominal surgery is accompanied by many such insults that place elders at particularly high risk for post-operative complications including fasting for gastrointestinal healing, addition of multiple drugs, immobility, nasogastric tubes, and bladder catheterization. Many of these are modifiable and attention to these risk factors should be assessed to prevent post-operative complications in this frail population. The results from this study underscore an important association between the development of complications and mortality. Therefore, this finding emphasizes the importance of implementing recommendations and best practices to prevent perioperative complications.

The present study is limited by its retrospective design, sample size, and recruitment from a single hospital. Understandably, only patients who could be included were those pre-selected by their surgeons for operative management, it is suspected that many elderly patients presenting to the emergency department with surgical disease that was managed non-operatively on non-surgical units or with end-of-life care goals.

Identifying patients at risk of developing in-hospital complications, and developing tailored preventative strategies, should be a focus to improve care for the elderly emergency general surgical patient. Age or number of comorbidies alone should not be the limiting factors for surgical referral or treatment. We advocate for the use of ASA class as both an available and robust tool for prediction of in-hospital mortality following emergency general surgery in very elderly patients. This information can be meaningful to patients and their families when used for perioperative counseling aimed at setting realistic expectations and assisting patients or surrogates decision makers to understand the goals of care and treatment.

## Abbreviations

ASA: American Society of Anesthesiologist Physical Status; BMI: Body mass index; CPS: Comorbidity-Polypharmacy score.

## Competing interests

The author(s) declare that they have no competing interests.

## Authors’ contributions

SM, RP, SW, RK contributed to study design. DH built a custom database for data acquisition. JP performed data acquisition, initial analysis, and wrote the initial draft manuscript. SM performed data analysis and wrote the final manuscript. All authors read and approved the final manuscript.
